# Spiramide and Hydroquinidine Inhibit Proliferation and Migration While Promoting Apoptosis and Oxidative Stress in Neuroblastoma Cells

**DOI:** 10.3390/ijms27146367

**Published:** 2026-07-17

**Authors:** Evren Gümüş, İlknur Keskin, Ezgi Yıldırım, Servet Kavak, Turan Demircan

**Affiliations:** 1 Medical Genetics Department, School of Medicine, Muğla Sıtkı Koçman University, Muğla 48000, Turkey; evrengumus@mu.edu.tr; 2 Histology and Embryology Department, School of Medicine, İstanbul Medipol University, İstanbul 34214, Turkey; 3 Molecular Biology and Genetics, Institute of Natural Sciences, Muğla Sıtkı Koçman University, Muğla 48000, Turkey; sci.ezgiyildirim@gmail.com; 4 Biophysics Department, School of Medicine, İzmir Bakırçay University, İzmir 35665, Turkey; servet.kavak@bakircay.edu.tr; 5 Medical Biology Department, School of Medicine, İzmir Bakırçay University, İzmir 35665, Turkey

**Keywords:** neuroblastoma, drug repurposing, hydroquinidine, AMI-193, apoptosis, cell proliferation, Reactive Oxygen Species (ROS), SH-SY5Y

## Abstract

Neuroblastoma is an aggressive pediatric malignancy with limited therapeutic options for high-risk disease, underscoring the need for alternative treatment strategies. Drug repurposing offers a promising approach to accelerate the identification of effective anti-cancer agents. In this study, we investigated the anti-carcinogenic effects of hydroquinidine, a class IA antiarrhythmic ion channel blocker, and spiramide, a dopamine D_2_/serotonin 5-HT_2_ receptor antagonist and endoplasmic reticulum stress inducer, in SH-SY5Y human neuroblastoma cells. Cells were treated with increasing concentrations of each compound and evaluated using cell viability, colony formation, wound healing, proliferation, apoptosis, and quantitative gene expression assays. Both compounds induced a dose-dependent reduction in cell viability, with spiramide exhibiting greater potency than hydroquinidine. Functional assays revealed significant suppression of clonogenic survival, cell migration, and DNA synthesis, accompanied by increased oxidative stress and cell death. Molecular analyses demonstrated coordinated transcriptional regulation of apoptosis- and cell cycle-related genes, characterized by upregulation of *BAX*, *CDKN1A*, and *CDKN1B*, and downregulation of *BCL-2* and *CCND1*. Notably, spiramide consistently produced stronger cytotoxic and wound-closure inhibitory effects, suggesting a greater contribution of oxidative stress- and apoptosis-associated pathways. Collectively, these findings indicate that hydroquinidine and spiramide disrupt neuroblastoma cell growth through complementary stress- and cell cycle-associated pathways and identify them as promising candidates for further preclinical evaluation.

## 1. Introduction

Neuroblastoma (NB) is an embryonal malignancy of the sympathetic nervous system and the most common extracranial solid tumor in young children [1]. It accounts for roughly 15% of all pediatric cancer-related deaths [2], reflecting the aggressive nature of high-risk cases. Despite intensive multimodal therapy, outcomes for high-risk neuroblastoma remain poor—five-year survival rates are only on the order of ~50–60% [3]. These statistics underscore an urgent need for novel therapeutic approaches, including the exploration of new drugs or the repurposing of existing drugs to improve neuroblastoma treatment.

In vitro studies of NB frequently employ the SH-SY5Y human neuroblastoma cell line as a model. SH-SY5Y cells are a thrice-subcloned subline derived from the SK-N-SH neuroblastoma line [4]. This cell line has been widely used for decades as an in vitro model in neurobiology and neuro-oncology research [4]. Its adaptability and capacity to exhibit neuronal characteristics upon differentiation make SH-SY5Y a useful system for testing potential anti-cancer agents in a neuroblastoma context.

Drug repurposing, the investigation of existing drugs for new therapeutic indications, offers a promising and accelerated path to identifying new cancer treatments. These compounds have already undergone extensive safety and pharmacokinetic testing, significantly reducing the time and cost associated with drug development [5]. The search for new agents often focuses on compounds that modulate pathways critical for cancer cell survival, such as ion channels, G-protein coupled receptors, and stress response mechanisms.

Hydroquinidine (HQ), a stereoisomer of quinidine, is a Class IA antiarrhythmic drug primarily used in cardiology to maintain normal sinus rhythm and treat conditions like Brugada syndrome [6]. Hydroquinidine’s anti-carcinogenic activity is hypothesized to stem from its primary function as an ion channel blocker [7]. Aberrant regulation of ion channels is increasingly recognized as a crucial contributor to tumorigenesis that intersects with and modulates multiple canonical hallmarks of cancer [8]. Rather than acting as a standalone canonical hallmark, ion channel dysfunction acts as an underlying pathophysiological driver that influences key tumor behaviors, including sustained proliferation, cell volume homeostasis during division, evasion of apoptotic signals, and active tissue invasion [8]. Studies have demonstrated that hydroquinidine exhibits significant anti-carcinogenic activity in several cancer types, including breast, ovarian, liver, glioblastoma, and colon cancer cells, by inhibiting cell-cycle progression and stimulating apoptosis [9,10,11]. These findings suggest that hydroquinidine exerts broad anticancer effects by interfering with tumor cell division and survival processes, warranting investigation of its impact in neuroblastoma.

Spiramide (AMI-193) is a diphenylbutylpiperidine derivative, chemically related to spiperone. While historically explored as an antipsychotic agent for schizophrenia, its pharmacological profile is characterized by high-affinity antagonism of the Dopamine D2 receptor and the Serotonin 5-HT2 receptor [12]. The D2 receptor is a significant target in cancer research, as its antagonists have been shown to reduce tumor growth and induce autophagy in various cancer models [13,14,15]. Furthermore, spiramide has been identified as an Endoplasmic Reticulum (ER) stress inducer [16]. ER stress, when prolonged and unresolvable, triggers the Unfolded Protein Response (UPR), which can ultimately lead to apoptosis in cancer cells [17]. This dual mechanism—modulating dopamine-related pathways and inducing cellular stress—positions spiramide as a promising candidate for drug repurposing in NB.

In this study, we examined the anti-cancer effects of spiramide and hydroquinidine on SH-SY5Y human neuroblastoma cells. We treated SH-SY5Y cells with these compounds and assessed multiple indicators of cancer cell behavior, including cell viability, clonogenic growth, migratory ability, and proliferation rates. We also evaluated the induction of apoptosis and changes in the expression of key genes involved in cell cycle regulation and apoptotic pathways. By integrating cell-based assays with gene expression profiling, our aim was to determine whether spiramide and hydroquinidine can inhibit neuroblastoma cell growth and to elucidate the potential mechanisms underlying their anti-carcinogenic effects.

## 2. Results

### 2.1. Dose-Dependent Effects of Hydroquinidine and Spiramide on Cell Viability

**Figure 1:**
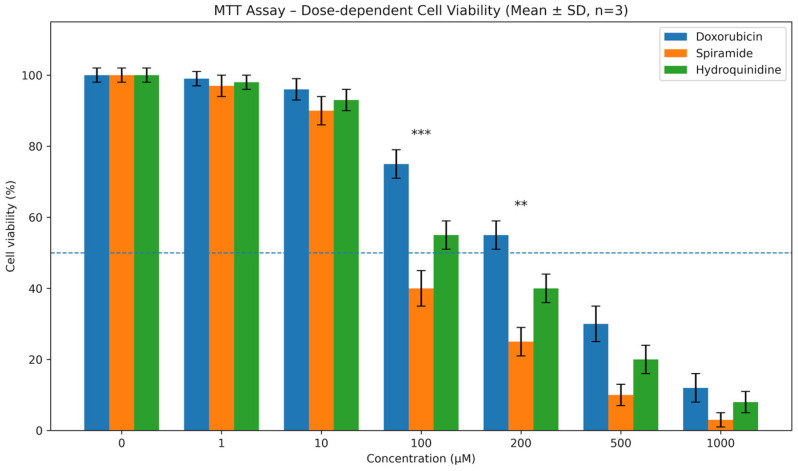
Dose-dependent effects of doxorubicin, spiramide, and hydroquinidine on cell viability determined by MTT assay. Cells were treated with increasing concentrations (0–1000 µM) for 24 h. Data are presented as mean ± SD of three independent experiments (*n* = 3). Statistical significance was assessed by one-way ANOVA test. The blue dashed line indicates 50% cell viability. ** *p* < 0.01; *** *p* < 0.001.

The cytotoxic effects of hydroquinidine and spiramide were evaluated using the MTT assay in SH-SY5Y cells over a broad concentration range (0–1000 µM). Both compounds induced a clear dose-dependent reduction in cell viability, albeit with markedly different potencies (Figure 1). Quantitative analysis revealed that spiramide exhibited the strongest cytotoxic activity, with an IC_50_ value of 50 µM. Consistent with this, cell viability declined to approximately 40% at 100 µM and fell below 10% at concentrations ≥ 500 µM. In contrast, hydroquinidine showed a more moderate cytotoxic profile, with an IC_50_ of 85 µM, corresponding to 55–60% viability at 100 µM and approximately 20% viability at 500 µM. Doxorubicin, included as a reference chemotherapeutic agent, displayed a delayed cytotoxic response with a substantially higher IC_50_ of 190 µM, maintaining cell viability above 70% at 100 µM and approaching 50% only at higher concentrations. Collectively, these results demonstrate a clear and differential potency of tested chemicals in reducing metabolic activity in SH-SY5Y cells.

### 2.2. Hydroquinidine and Spiramide Suppress Long-Term Clonogenic Survival

**Figure 2:**
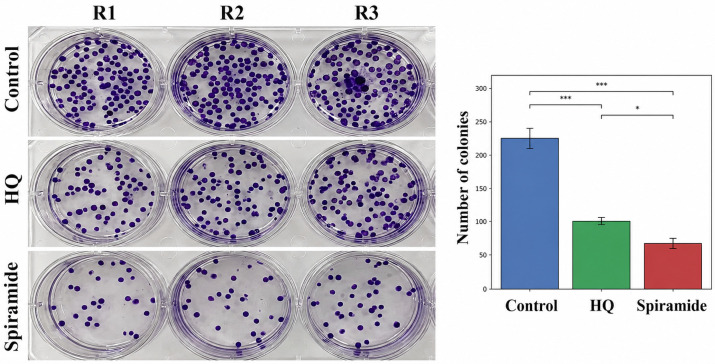
Effects of hydroquinidine and spiramide on clonogenic survival of SH-SY5Y neuroblastoma cells. Cells were treated at their respective 24 h IC_50_ concentrations for a continuous duration of 10 days before crystal violet staining and automated colony counting. Data are presented as mean ± SD of three independent experiments (*n* = 3). Statistical analysis was performed using one-way ANOVA followed by Tukey’s post-hoc test. * *p* < 0.05; *** *p* < 0.001.

To determine whether the observed reduction in metabolic activity translated into impaired long-term proliferative capacity, a colony formation assay was performed under continuous exposure to each compound at their respective 24 h IC_50_ over an incubation period of 10 days (Figure 2). Control cells formed a high number of colonies, with an average of 225 ± 15 colonies per well. Treatment with hydroquinidine significantly reduced clonogenic survival, resulting in approximately 100 ± 5 colonies, corresponding to a reduction of nearly 55% compared to control (*p* < 0.001). Spiramide exerted an even stronger effect, reducing colony numbers to 67 ± 8 colonies, representing a ~70% decrease relative to the control (*p* < 0.001). Moreover, spiramide significantly suppressed colony formation compared to hydroquinidine (*p* < 0.05). These data indicate that both compounds compromise long-term clonogenic survival, with spiramide displaying superior inhibitory efficacy.

### 2.3. Reduced Wound Closure in Hydroquinidine- and Spiramide-Treated Cells

**Figure 3:**
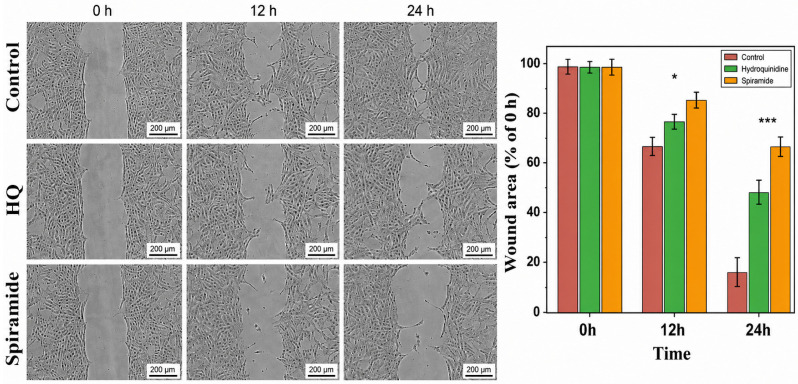
Effects of hydroquinidine and spiramide on wound healing of SH-SY5Y cells as assessed by scratch assay. Wound area was quantified at 0, 12, and 24 h and expressed as percentage of the initial wound area (0 h). Data are presented as mean ± SD of three independent experiments (*n* = 3). * *p* < 0.05; *** *p* < 0.001.

The effects of hydroquinidine and spiramide on cell migratory capacity were assessed using a wound healing assay (Figure 3). At baseline (0 h), wound area was normalized to 100% for all groups. Control cells exhibited rapid wound closure, reducing the wound area to approximately 65 ± 4% at 12 h and 15 ± 6% at 24 h. In contrast, hydroquinidine-treated cells showed significantly delayed migration, with wound areas of 75 ± 3% at 12 h and 45 ± 5% at 24 h (*p* < 0.05 at 12 h; *p* < 0.001 at 24 h versus control). Spiramide treatment resulted in the most pronounced inhibition of migration, with wound areas remaining at 85 ± 3.5% at 12 h and 65 ± 4% at 24 h (*p* < 0.001 versus control at both time points). These findings indicate that both compounds reduce wound closure, which may reflect a combination of impaired migration, reduced proliferation, and increased cytotoxic stress, particularly under IC_50_-level exposure.

### 2.4. Reduced Proliferative Activity Confirmed by EdU Incorporation

**Figure 4:**
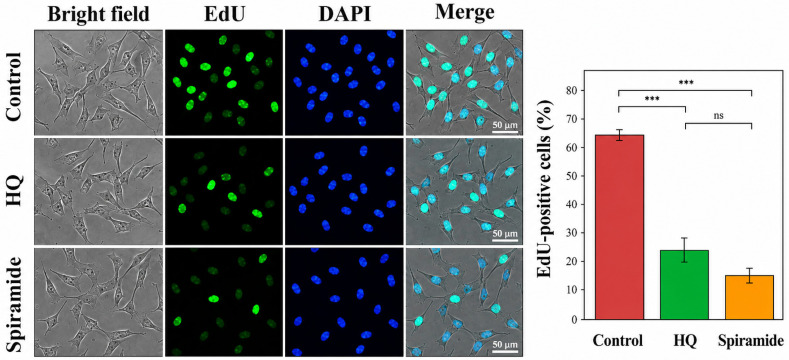
Effects of hydroquinidine and spiramide on cell proliferation assessed by EdU incorporation assay. The percentage of EdU-positive SH-SY5Y cells was quantified and expressed as mean ± SD of three independent experiments (*n* = 3). Statistical analysis was performed using one-way ANOVA followed by Tukey’s post-hoc test. *** *p* < 0.001; ns, not significant.

To further investigate the impact of hydroquinidine and spiramide on cell proliferation, EdU incorporation was analyzed as a direct measure of DNA synthesis (Figure 4). Control cells displayed a high proportion of EdU-positive nuclei (65.0 ± 1.7%), reflecting active proliferation. Hydroquinidine treatment significantly reduced the percentage of EdU-positive cells to 25.0 ± 4.9% (*p* < 0.001 versus control), while spiramide reduced EdU incorporation to 15.0 ± 3.0% (*p* < 0.001 versus control).

Notably, despite the numerically lower proliferation rate observed with spiramide, no statistically significant difference was detected between hydroquinidine and spiramide (*p* > 0.05). This suggests that both compounds comparably suppress DNA synthesis and cell cycle progression.

### 2.5. Hydroquinidine and Spiramide Induce a Robust G0/G1 Phase Arrest in SH-SY5Y Cells

**Figure 5:**
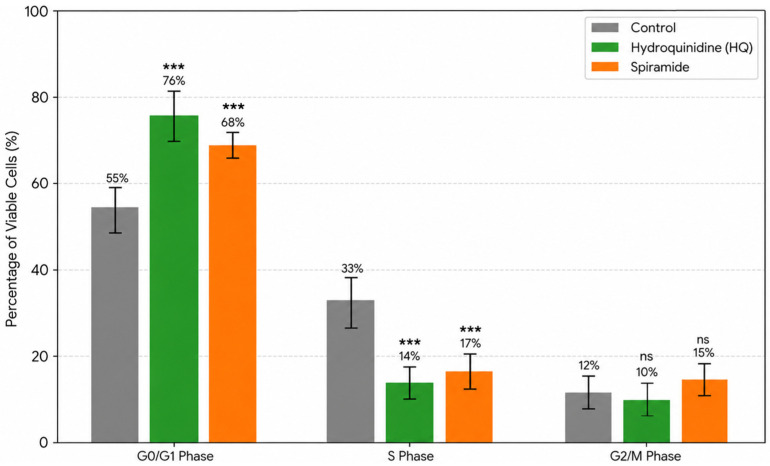
Quantitative cell cycle distribution of viable SH-SY5Y neuroblastoma cells following 24 h exposure to hydroquinidine (85 μM) or spiramide (50 μM). Sub-G1 debris fractions were excluded from gating to normalize the viable cell cycle distribution to 100%. Independent biological replicates are illustrated via mean percentages (±SD, *n* = 3). Inter-group statistical differences compared to respective controls were calculated via one-way ANOVA followed by Tukey’s post-hoc test (*** *p* < 0.001; ns: non-significant).

To clarify whether the observed suppression of cell viability and depletion of active DNA synthesis were mediated by specific cell cycle checkpoints, we quantified the distribution of viable neuroblastoma cells across the G0/G1, S and G2/M phases via PI flow cytometry (Figure 5). The untreated control population showed a typical proliferative cell cycle profile, with 55 ± 4.24% of cells residing in the G0/G1 phase, 33 ± 5.10% in the active S phase, and 12 ± 3.11% in the mitotic G2/M phase.

Exposure to hydroquinidine (85 μM) for 24 h induced a significant accumulation of cells in the G0/G1 phase, increasing to 76 ± 5.35% (*p* < 0.001). This checkpoint arrest occurred at the expense of the replicating compartment, where the S phase population dropped significantly to 14 ± 3.20% (*p* < 0.001), while the G2/M fraction remained statistically unchanged (10 ± 3.12%, ns).

Spiramide (50 μM) treatment similarly induced marked G0/G1 arrest, with 68 ± 3.42% of viable cells accumulating in this phase (*p* < 0.001). Mirroring the hydroquinidine pattern, spiramide triggered a robust contraction of the actively replicating S phase population down to 17 ± 3.65% (*p* < 0.001), with no significant change in the G2/M phase (15 ± 3.48%, ns). Collectively, these results indicate that both compounds induce cell cycle arrest at the G1/S checkpoint in neuroblastoma cells.

### 2.6. Hydroquinidine and Spiramide Increase Cell Death

**Figure 6:**
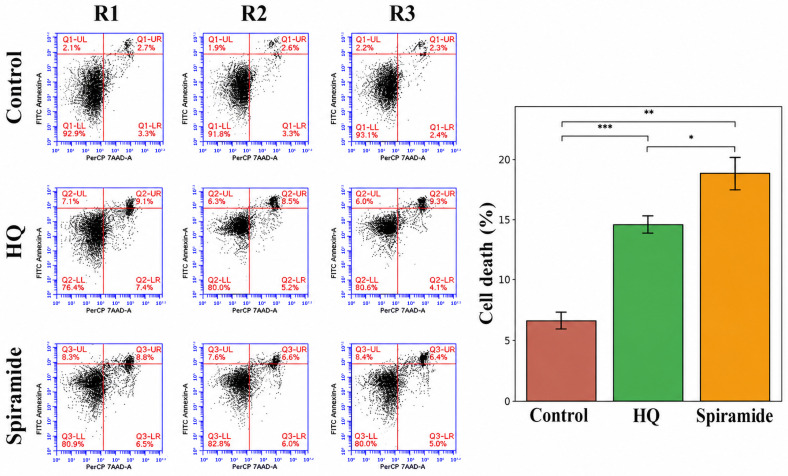
Hydroquinidine and spiramide increase cell death in SH-SY5Y cells. Representative Annexin V-FITC/PI flow cytometry dot plots and quantification of total (non-viable) cell fraction following 24 h treatment at respective IC_50_ concentrations. A minimum of 10,000 single-cell events were acquired per sample. Data are presented as mean ± SD of three independent experiments (*n* = 3). Statistical analysis was performed using one-way ANOVA followed by Tukey’s post-hoc test. * *p* < 0.05; ** *p* < 0.01; *** *p* < 0.001.

As a next step, to quantify the extent of cell death induced by each compound, SH-SY5Y cells were stained with Annexin V-FITC/PI and analyzed by flow cytometry following 24 h treatment at their respective IC_50_ concentrations (Figure 6). Total cell death was defined as the combined fraction of non-viable cells (Annexin V^+^ and/or PI^+^ events, encompassing early apoptotic, late apoptotic, and necrotic populations).

Control cells exhibited a low basal level of cell death (6.53 ± 0.61%). Hydroquinidine treatment significantly increased cell death to 14.43 ± 0.67% (*p* < 0.001 versus control), while spiramide induced the highest level of cell death, reaching 18.77 ± 1.43%, which was significantly higher than both control (*p* < 0.01) and hydroquinidine-treated cells (*p* < 0.05). These findings indicate that both compounds promote cell death, with spiramide exerting a more pronounced cytotoxic effect.

### 2.7. Intracellular Reactive Oxygen Species (ROS) Accumulation

**Figure 7:**
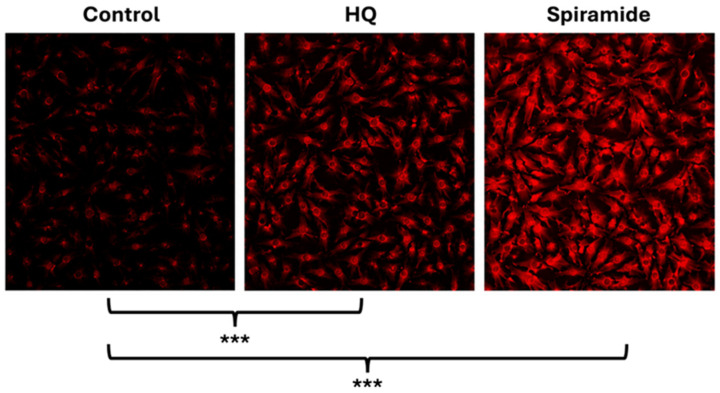
Hydroquinidine and spiramide increase intracellular ROS accumulation in SH-SY5Y cells. Representative fluorescence microscopy images of SH-SY5Y cells stained with ROS Brite™ DHCF. Untreated control cells show weak basal fluorescence. Hydroquinidine-treated cells display elevated ROS-associated signal, while spiramide treatment elicits the strongest fluorescence intensity demonstrating a treatment-dependent escalation in oxidative stress. Images were acquired using a 10× objective. *** *p* < 0.001.

To evaluate intracellular ROS accumulation in SH-SY5Y cells, ROS Brite™ DHCF staining was performed, with representative fluorescence images shown in Figure 7. Untreated control cells exhibited relatively weak, sparsely distributed fluorescence, indicating a low basal level of intracellular oxidative activity and minimal background ROS production under standard culture conditions. In contrast, both hydroquinidine and spiramide treatments triggered a robust, statistically significant elevation in intracellular ROS levels compared to the control group (*p* < 0.001). Quantitative analysis of fluorescence intensity revealed a 3.4-fold increase in hydroquinidine-treated cells and a 5.1-fold increase in spiramide-treated cells relative to the control.

Hydroquinidine administration resulted in a marked increase in ROS-associated signal intensity and a wider distribution throughout the cell population. This induction was characterized by a visibly higher frequency of fluorescence-positive cells and intensified cytoplasmic staining, demonstrating enhanced intracellular ROS generation. Among the experimental groups, spiramide elicited the most prominent oxidative response. Spiramide-treated SH-SY5Y cells displayed intense, diffuse fluorescence across the vast majority of the population. These findings suggest that ROS accumulation may contribute to spiramide-mediated cytotoxicity and apoptosis in SH-SY5Y cells.

### 2.8. Regulation of Apoptosis- and Cell Cycle-Related Genes by Hydroquinidine and Spiramide in SH-SY5Y Cells

**Figure 8:**
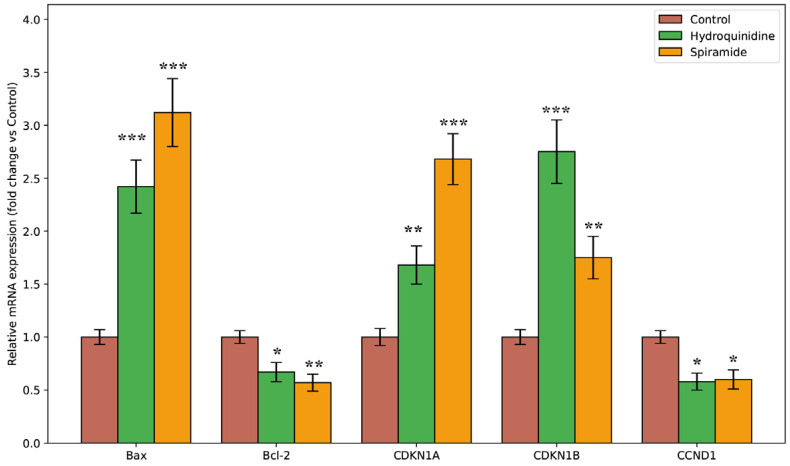
Representative gene expression changes induced by hydroquinidine and spiramide. Relative mRNA expression levels of *BAX*, *BCL-2*, *CDKN1A*, *CDKN1B*, and *CCND1* were quantified by qPCR following treatment with hydroquinidine or spiramide. Gene expression levels were normalized to the control group and are presented as fold change relative to control. Data represent mean ± SD from three independent experiments (*n* = 3). * *p* < 0.05, ** *p* < 0.01, and *** *p* < 0.001.

Quantitative real-time PCR (qPCR) analysis revealed that both hydroquinidine and spiramide markedly altered the expression of apoptosis- and cell cycle-related genes (Figure 8). Compared to the control (normalized to 1.0), the pro-apoptotic gene Bax was upregulated to 2.42 and 3.12 following hydroquinidine and spiramide treatment, respectively, whereas the anti-apoptotic gene Bcl-2 was downregulated to 0.67 and 0.57. Analysis of cell cycle regulators showed increased expression of CDKN1A, reaching 1.68 in hydroquinidine- and 2.68 in spiramide-treated cells, and CDKN1B, which was more strongly induced by hydroquinidine (2.75) than spiramide (1.75). In contrast, expression of CCND1 was reduced in both treatment groups (0.58 for hydroquinidine and 0.60 for spiramide). These results indicate coordinated transcriptional activation of apoptotic pathways and suppression of cell cycle progression following treatment with both compounds.

## 3. Discussion

The present study provides the first comparative evaluation of the antipsychotic spiramide (AMI-193) and the antiarrhythmic hydroquinidine as potential therapeutic agents for high-risk neuroblastoma. Our findings demonstrate that both compounds exert significant dose-dependent cytotoxicity against SH-SY5Y cells, with spiramide displaying superior potency (IC_50_ 50 µM) compared to hydroquinidine (IC_50_ 85 µM). Notably, under the present 24 h assay conditions, spiramide and hydroquinidine yielded lower calculated IC_50_ values than doxorubicin.

The pronounced anti-neoplastic activity of spiramide observed in this study aligns with the “neuro-oncological” hypothesis, which posits that neuroblastoma cells rely on autocrine catecholamine signaling for survival [14,18]. Spiramide is a potent antagonist of Dopamine D2 and Serotonin 5-HT2 receptors [19]. Previous studies have established that dopamine antagonists can inhibit macromolecular synthesis and tumor growth in neuroblastoma xenografts by disrupting autocrine dopaminergic loops [20,21,22]. Our data reinforce this, showing that spiramide treatment not only reduced metabolic viability but also drastically impaired clonogenic survival (~70% reduction) and cell migration. Spiramide-treated cells showed minimal wound closure even after 24 h, indicating a substantial impediment to cell motility. This could result from cytoskeletal stabilization or interference with motility-related signaling. Dopamine D_2_ receptors have been reported to cross-talk with pathways that regulate the cytoskeleton and cell adhesion; their blockade might reduce the dynamic cell movements required for migration [23,24]. While the exact molecular targets for the anti-migratory action remain to be determined, our data clearly indicate that spiramide reduces the invasive potential of neuroblastoma cells, which is encouraging for therapies aimed at containing tumor spread.

Moreover, spiramide was identified in a high-throughput screen as an inducer of ER stress [16]. In that report, it was demonstrated that spiramide and spiperone activate all three branches of the UPR—PERK/eIF2α phosphorylation, ATF6, and IRE1/XBP1 splicing—hallmarks of activation [16]. Inducing ER stress beyond a critical threshold can tip cancer cells into apoptosis, especially in tumors that are heavily reliant on ER and proteasomal function for their high protein turnover [25,26]. In our experiments, we did not measure UPR markers directly; however, the downstream consequences of severe ER stress would be consistent with what we observed. The molecular analysis revealed a robust upregulation of *BAX* (3.12-fold) and *CDKN1A* (p21, 2.68-fold) accompanied by *BCL-2* suppression. UPR-mediated transcription factors (like CHOP) can upregulate *CDKN1A* (p21) and Bax while downregulating pro-survival factors, driving cell-cycle arrest and apoptotic cell death [27,28]. This proposed transcriptional framework is consistent with our functional flow cytometric findings, in which spiramide treatment led to a marked accumulation of viable cells in the G0/G1 phase (68%) alongside a corresponding reduction in the S-phase population (17%) (Figure 5), although direct confirmation of UPR pathway activation was not performed in this study. Thus, spiramide’s known action as an ER stress inducer, combined with dopamine/serotonin receptor antagonism, provides a plausible explanation for its superior efficacy. This dual mechanism—receptor blockade (D_2_R) together with ER stress/UPR activation—may account for its stronger anti-carcinogenic effect.

Oxidative stress results demonstrate that both hydroquinidine and spiramide significantly elevate intracellular ROS accumulation in SH-SY5Y neuroblastoma cells, with spiramide eliciting the most robust response. Exceeding the oxidative stress threshold that a cancer cell can handle may trigger a cascade of mitochondrial dysfunction, DNA damage, and cell-cycle arrest, shifting cells from adaptive survival to irreversible apoptosis [29]. Therefore, the pronounced ROS accumulation observed here likely represents a key mechanism driving the anticarcinogenic activity of these compounds rather than a generic, non-specific cytotoxic artifact. Our findings support the premise that hydroquinidine-mediated growth inhibition in SH-SY5Y cells is driven, at least in part, by the activation of oxidative stress-dependent death pathways, while spiramide’s action likely stems from the pharmacological targeting of serotonergic and dopaminergic axes known to disrupt mitogenic signaling and reduce tumor viability.

Exploiting oxidative stress vulnerabilities has been increasingly explored as a strategy to overcome chemoresistance in neuroblastoma and other malignancies. For instance, recent studies on alternative pro-oxidant agents in SH-SY5Y models demonstrate that the novel aroma compound Biosacetalin (1,1-Diethoxyethane) induces apoptosis by inhibiting mitochondrial Complex I, leading to rapid intracellular ROS accumulation [30]. Furthermore, while severe, excessive ROS accumulation can trigger mitochondrial dysfunction and dysregulated mitophagy, cells often attempt to counteract this damage via endogenous defense pathways. This is evidenced by findings where the lipid peroxidation inhibitor Liproxstatin-1 successfully rescued SH-SY5Y cells from mitochondrial-mediated intrinsic apoptosis by suppressing severe oxidative membrane damage [31]. Taken together, the concurrent induction of apoptosis and growth inhibition by hydroquinidine and spiramide is compatible with a scenario in which the observed ROS accumulation overwhelms endogenous antioxidant defense mechanisms, thereby contributing to the commitment of neuroblastoma cells to apoptotic cell death. Direct assessment of mitochondrial function and antioxidant pathway activity would be required to substantiate this interpretation.

Likewise, hydroquinidine demonstrated significant anti-carcinogenic properties. Our data on hydroquinidine’s anti-neuroblastoma activity resonate with reports in other tumor models. Hydroquinidine is a stereoisomer of quinidine and a known Class IA antiarrhythmic (used, for example, in Brugada syndrome patients), but emerging evidence indicates it has broad antineoplastic properties [9,10,11]. In breast, ovarian, glioblastoma, and lung cancer cells, hydroquinidine was previously shown to inhibit growth by inducing cell-cycle arrest and apoptosis. Moreover, it has been reported that hydroquinidine decreased colony formation and migration of A549 lung carcinoma cells while downregulating genes governing cell division/survival and upregulating those promoting cell-cycle arrest and apoptosis [32]. This mirrors our observations in SH-SY5Y cells, suggesting a conserved mechanism of action across cancer types.

The antiproliferative effects of hydroquinidine are likely linked to its primary pharmacological role as an ion channel blocker. Ion channels are increasingly recognized as important enablers of cancer cell proliferation, migration, and survival [33,34]. Dysregulated ion flux (e.g., through potassium, sodium, and calcium channels) is a common characteristic of malignancies, contributing to uncontrolled growth, metastatic behavior and resistance to stress signals [8,35]. By blocking such channels, hydroquinidine may disrupt the ionic homeostasis and signaling that neuroblastoma cells rely on, thereby triggering stress responses and growth arrest. Indeed, hydroquinidine likely disrupts the precise ion fluxes required for cell volume regulation during the cell cycle. The significant upregulation of *CDKN1B* (p27, 2.75-fold) observed in hydroquinidine-treated cells is of particular interest. Unlike spiramide, which predominantly induced CDKN1A, HQ preferentially induced CDKN1B (p27), suggesting a potentially distinct mechanism of G0/G1 arrest. Based on prior reports, this may be related to altered membrane potential or ion flux required for the G1/S transition [36]; however, it must be explicitly noted that our study does not provide direct experimental proof for this specific causal link. Although less potent than spiramide in long-term clonogenic survival, hydroquinidine’s capacity to alter the BAX/BCL-2 ratio and suppress *CCND1* expression supports a role in regulating tumor cell cycle progression, though this requires further electrophysiological confirmation.

The significant inhibition of migration by hydroquinidine (45% wound gap at 24 h) is in line with the emerging onco-channelopathy framework, in which dysregulated ion transport has been proposed to support tumor invasion by influencing cytoskeletal organization and cell volume dynamics required for cell motility [37,38]. These observations suggest that targeting ion channels may affect multiple biophysical processes underlying tumor spread, rather than a single canonical hallmark [37]. Hydroquinidine’s significant anti-migratory effect in our wound-healing assay could stem from impairing ion channel-regulated processes like cell volume dynamics or motility signaling [37,39]—consistent with the anti-migratory action of hydroquinidine noted in other models. An additional morphological observation during the wound healing assay was a localized widening of the scratch boundary in spiramide-treated monolayers at 24 h. This may reflect a dual effect of spiramide at the wound edge: impaired directional migration together with increased cell death among cells at the leading edge, which may be more vulnerable due to reduced cell–cell contact. This interpretation is consistent with our flow cytometric data showing increased apoptosis and a 5.1-fold increase in ROS levels in spiramide-treated cells; however, cell viability was not directly assessed at the wound margin itself, and this explanation should be regarded as hypothesis-generating rather than conclusive.

A critical consideration when evaluating the translational potential of these findings is that the acute 24 h in vitro IC_50_ values are considerably lower than what may be clinically achievable without systemic toxicity. Pushing these agents to such high systemic concentrations clinically risks severe off-target toxicities, including QTc prolongation/pro-arrhythmias for hydroquinidine and profound neurological side effects via dopamine D_2_/serotonin 5-HT_2_ antagonism for spiramide. However, these thresholds must be carefully contextualized. Due to their lipophilic structures, both compounds possess high tissue distribution tendencies, potentially leading to elevated localized concentrations within solid tumor masses compared to peripheral plasma. Furthermore, our long-term colony formation assays confirmed that continuous, extended exposure achieves significant clonogenic suppression at much lower thresholds. Ultimately, to bypass systemic toxicity while exploiting their anti-neuroblastoma efficacy, future strategies should avoid high-dose single-agent interventions. Instead, translational efforts must focus on low-dose synergistic combinations with standard-of-care chemotherapeutics or the deployment of advanced targeted nanoformulations designed to selectively deliver high drug concentrations directly into the tumor microenvironment while sparing healthy tissues.

A limitation of this study is that the expression of the apoptosis- and cell cycle-related genes (*BAX*, *BCL-2*, *CCND1*, *CDKN1A*, and *CDKN1B*) was assessed only at the mRNA level. Although these transcriptional changes are consistent with our functional findings—reduced EdU incorporation, impaired clonogenic growth, and increased Annexin V staining—mRNA levels do not always correlate directly with protein expression. Future work should therefore confirm these findings at the protein level, for example by Western blotting or immunofluorescence for BAX, BCL-2, Cyclin D1, p21, and p27, and by assessing cleaved caspase-3, caspase-9, and PARP to clarify the apoptotic pathway involved. Rescue experiments using the pan-caspase inhibitor z-VAD-FMK would also help determine whether hydroquinidine- and spiramide-induced cytotoxicity is caspase-dependent. In addition, the proposed mechanisms remain indirect: UPR activation was not measured directly and should be assessed via GRP78, CHOP, and XBP1 splicing in spiramide-treated cells, while patch-clamp analysis of ion channel activity would help identify the specific targets of hydroquinidine. Since doxorubicin showed only modest activity in our viability assay, combining hydroquinidine or spiramide with low-dose chemotherapy may offer a promising strategy to enhance efficacy while reducing systemic toxicity. Finally, multi-omics and network pharmacology approaches [40,41], together with nanoformulation strategies and in vivo models [42], will be important next steps toward translating these findings into clinical application.

Given the persistently poor outcomes in high-risk neuroblastoma [2], the discovery of such pronounced anti-tumor effects from repurposed compounds is noteworthy. While further research is necessary, our findings open new perspectives on leveraging ion channel blockers and dopamine/serotonin antagonists as novel therapeutic strategies in neuroblastoma.

## 4. Materials and Methods

### 4.1. Cell Culture Conditions

The human neuroblastoma cell line SH-SY5Y (ATCC-CRL-2266) was cultured in Dulbecco’s Modified Eagle’s Medium (DMEM; Sigma-Aldrich, Darmstadt, Germany, cat. no. D6429) supplemented with 10% fetal bovine serum (FBS; Thermo Fisher Scientific, Waltham, MA, USA, cat. no. A4736401) and 1% penicillin–streptomycin solution (Thermo Fisher Scientific, cat. no. 15140122). Cells were maintained at 37 °C in a humidified atmosphere containing 5% CO_2_ and routinely passaged upon reaching approximately 80% confluency. Cell morphology and growth were regularly monitored using an inverted light microscope (SOPTOP ICX41, Ningbo, China).

Hydroquinidine, spiramide and doxorubicin stock solutions were prepared at 100 mM in cell culture-grade dimethyl sulfoxide (DMSO; PAN-Biotech, Aidenbach, Germany, cat. no. P60-36720100) and stored according to manufacturer recommendations. Working concentrations were freshly generated by serial dilution in complete culture medium immediately prior to use. At the highest tested concentration (1 mM), the final DMSO concentration did not exceed 1% (*v*/*v*), a level previously shown to be non-cytotoxic. Control cells received complete culture medium containing the corresponding final concentration of DMSO used in the treated wells. All experiments were performed independently in triplicate (*n* = 3).

### 4.2. Cell Viability Assay

Cell viability was determined using the CellTiter 96^®^ Non-Radioactive Cell Proliferation Assay (Promega, Fitchburg, WI, USA, cat. no. G4000). SH-SY5Y cells were seeded into 96-well plates at a density of 1 × 10^4^ cells per well in 100 µL of complete medium and allowed to adhere for 24 h. Cells were then treated with hydroquinidine or spiramide across a concentration range of 0.0001–1 mM for 24 h using a single-dose exposure protocol. Untreated wells served as negative controls, whereas 10% DMSO was used as a positive cytotoxic control. Following treatment, the dye solution was added and incubated for 4 h, after which the solubilization/stop solution was applied to terminate the reaction. Absorbance was measured at 570 nm using SpectraMax i3 (Molecular Devices, Silicon Valley, CA, USA) microplate reader. Half-maximal inhibitory concentration (IC_50_) values were calculated by nonlinear regression analysis of dose–response curves using the “drc” package in R (Version 4.5.3) [9].

### 4.3. Colony Formation Assay

The clonogenic capacity of SH-SY5Y cells following compound exposure was assessed using a colony formation assay. Cells were seeded at a density of 5 × 10^2^ cells per well in 6-well plates containing 1 mL of complete medium. Cells were continuously treated with hydroquinidine or spiramide at concentrations corresponding to the 24 h IC_50_ values determined in the viability assay, while control wells received drug-free medium. Culture medium was replaced every 48 h throughout the assay period, which continued until control cells reached ≥80% confluency. Colonies were fixed with 100% methanol for 20 min at room temperature, stained with 0.2% crystal violet for 15 min, and gently washed twice with distilled water to remove excess stain. Plates were air-dried, and colony images were acquired using a bright-field microscope (SOPTOP ICX41, Ningbo, China). Quantitative analysis of colony number and intensity was performed using the ColonyArea plugin in ImageJ software (Version 1.54p) [10].

### 4.4. Wound Healing Assay

Cell migratory capacity was evaluated using a scratch wound healing assay. SH-SY5Y cells were plated in 24-well plates at a density of 1 × 10^5^ cells per well and cultured for 24 h to allow monolayer formation. Cells were then treated with hydroquinidine or spiramide at their respective 24 h IC_50_ concentrations, while control wells received untreated medium. A uniform wound was created in each well using a sterile 200 µL pipette tip. Images of the wound area were captured immediately (0 h) and after 12 and 24 h using a bright-field microscope (SOPTOP ICX41). Quantification of wound closure was performed with the MRI_Wound_Healing_Tool plugin in ImageJ [11].

### 4.5. Proliferation Assay

Cell proliferation was examined using the Click-iT™ EdU Cell Proliferation Kit (Thermo Fisher Scientific, cat. no. C10337). SH-SY5Y cells were seeded into 96-well plates at a density of 1 × 10^4^ cells per well and incubated overnight. Cells were treated with hydroquinidine or spiramide at their 24 h IC_50_ concentrations, while untreated cells served as controls. No positive proliferation control was included. After 24 h of treatment, EdU solution (10 µL per well) was added and cells were incubated for an additional 2 h. Cells were then fixed with 100% methanol, washed with bovine serum albumin in phosphate-buffered saline (PBS), and permeabilized using a saponin-based reagent. EdU incorporation was detected using the Click-iT^®^ reaction cocktail according to the manufacturer’s protocol. Nuclei were counterstained with Hoechst dye, and fluorescence images were obtained using a Nikon Eclipse Ts2 microscope (Nikon Instruments Inc., Tokyo, Japan). Quantification of EdU-positive and total nuclei was performed using ImageJ software.

### 4.6. Cell Cycle Analysis by Flow Cytometry

To evaluate the precise distribution of cells across distinct cell cycle phases, PI DNA staining was performed. SH-SY5Y cells (1 × 10^5^ cells/well) were seeded in 12-well plates and incubated for 24 h to ensure monolayer attachment. The culture medium was then replaced with either control medium or medium containing hydroquinidine 85 µM or spiramide 50 µM at their respective 24 h IC_50_ thresholds, and the treatment was maintained for an additional 24 h. Following the treatment window, cells were harvested, washed with ice-cold PBS, and fixed in 70% cold ethanol for 30 min at 4 °C. Fixed cells were washed twice with PBS and treated with 50 µL of a 100 µg/mL RNase A solution (Sigma-Aldrich) for 30 min at 37 °C to eliminate RNA-associated background signals. Cellular DNA was subsequently counterstained by adding 200 µL of a 50 µg/mL PI solution. A minimum of 10,000 single-cell events were acquired per independent biological replicate (n = 3) using a BD Accuri™ C6 Plus flow cytometer (BD Biosciences, Paramus, NJ, USA). To evaluate authentic phase-specific somatic growth arrest and rule out mathematical skewing from dead cell remnants, the sub-G1 (apoptotic debris) population was excluded during analytical gating, and the viable cell cycle distribution was normalized to 100% using FlowJo software (Version 10.10).

### 4.7. Cell Death Analysis by Annexin V/PI Staining

Apoptotic cell death was assessed using the Alexa Fluor^®^ 488 Annexin V/Dead Cell Apoptosis Kit (Thermo Fisher Scientific, cat. no. V13242). SH-SY5Y cells were seeded at 1 × 10^5^ cells per well in 12-well plates and incubated for 24 h. Cells were subsequently treated with hydroquinidine or spiramide at their 24 h IC_50_ concentrations or left untreated as controls. After 24 h of exposure, cells were collected, washed with PBS, and resuspended in 100 µL of 1× annexin-binding buffer. FITC-conjugated Annexin V and propidium iodide (PI) were added according to the manufacturer’s instructions, followed by incubation for 15 min at room temperature in the dark. Samples were diluted with 400 µL annexin-binding buffer and analyzed by flow cytometry using a BD Accuri™ C6 Plus instrument, collecting exactly 10,000 single-cell events per independent biological replicate for subsequent population gating. Total cell death was calculated as the sum of all non-viable populations (Annexin V^+^/PI^−^, Annexin V^+^/PI^+^, and Annexin V^−^/PI^+^ events), excluding the viable (Annexin V^−^/PI^−^) cell fraction.

### 4.8. Detection of Intracellular ROS Levels

Intracellular ROS levels were evaluated using the fluorogenic probe ROS Brite™ DHCF (AAT Bioquest, Pleasanton, CA, USA, Cat#16053). A stock solution of the probe was prepared in DMSO and freshly diluted in serum-free medium to achieve a final working concentration of 5 µM. SH-SY5Y cells were seeded into 96-well plates at a density of 1 × 10^4^ cells/well and allowed to adhere overnight under standard culture conditions. Cells were then treated with hydroquinidine or spiramide at their respective 24-h IC_50_ concentrations; untreated cells served as the negative control group. Following a 24-h incubation period, the treatment medium was removed, and cells were incubated with the 5 µM ROS Brite™ DHCF working solution for 30 min at 37 °C in the dark. Subsequently, the staining solution was aspirated, and the cells were gently washed with PBS to remove excess extracellular probe. Fluorescence images were captured immediately using a Nikon Eclipse Ts2 microscope. Quantitative measurements were conducted via software-assisted fluorescence intensity analysis. Exposure time, gain, and all other acquisition settings were kept identical across all experimental groups to ensure accurate comparative analysis.

### 4.9. Gene Expression Analysis

Quantitative real-time PCR (qRT-PCR) was conducted to evaluate transcriptional changes in selected proto-oncogenes and tumor suppressor genes. The selected gene expression panel (*BAX*, *BCL-2*, *CDKN1A*, *CDKN1B*, and *CCND1*) was chosen based on a structured, hypothesis-driven approach to directly evaluate the molecular counter-responses associated with our phenotypic proliferation and cell death readouts, minimizing selection bias by covering both executioner and regulatory arms of cell homeostasis. SH-SY5Y cells were seeded at 3 × 10^5^ cells per well in 6-well plates and incubated for 24 h. Cells were then treated with hydroquinidine or spiramide for an additional 24 h, while control cells received fresh culture medium. Total RNA was isolated using a previously described protocol [11]. Extracted RNA was reverse-transcribed into complementary DNA (cDNA), and qRT-PCR was performed using gene-specific primers. GAPDH was used as an internal reference gene, and relative expression levels were calculated using the 2^−ΔΔCt^ method.

### 4.10. Statistical Analyses

All data are presented as mean ± standard deviation (SD) from three independent experiments, unless otherwise stated. Data normality was assessed using the Shapiro–Wilk test. Comparisons among multiple treatment groups were performed using one-way analysis of variance (ANOVA) followed by Tukey’s post hoc multiple-comparison test. For wound healing assays, the effects of treatment, time, and their interaction were analyzed using two-way ANOVA followed by Tukey’s post hoc test. Dose–response curves and IC_50_ values were estimated by non-linear regression analysis using the drc package in R. Statistical analyses were performed using the rstatix package in R, and graphical representations were generated in R. A *p*-value < 0.05 was considered statistically significant.

## 5. Conclusions

In conclusion, our study shows that both spiramide and hydroquinidine suppress SH-SY5Y neuroblastoma cell growth by inducing cell cycle arrest and apoptosis, with spiramide consistently showing greater potency—particularly in reducing cell viability, clonogenic survival, and cell migration. These effects were accompanied by coordinated changes in apoptosis- and cell cycle-related gene expression, including upregulation of the pro-apoptotic gene *BAX* and the cyclin-dependent kinase inhibitors *CDKN1A* and *CDKN1B*, alongside downregulation of *BCL-2* and *CCND1*. These findings support the potential of repurposing hydroquinidine and spiramide for neuroblastoma treatment and identify spiramide, in particular, as a promising candidate for further preclinical evaluation.

## Data Availability

The original contributions presented in this study are included in the article. Further inquiries can be directed to the corresponding authors.

## Abbreviations

The following abbreviations are used in this manuscript:

5-HT2	5-hydroxytryptamine
Bcl-2	B-cell lymphoma 2
CCND1	Cyclin D1
CDKN1A	Cyclin Dependent Kinase Inhibitor 1A
CDKN1B	Cyclin Dependent Kinase Inhibitor 1B
D_2_R	Dopamine receptor D_2_
EdU	5-ethynyl-2′-deoxyuridine
ER	Endoplasmic Reticulum
HQ	Hydroquinidine
IC_50_	Half-Maximal Inhibitory Concentration
NB	Neuroblastoma
qRT-PCR	Quantitative real-time PCR
SD	Standard Deviation
UPR	Unfolded Protein Response
ROS	Reactive Oxygen Species
